# Thy-1, a Pathfinder Protein for the Post-genomic Era

**DOI:** 10.3389/fcell.2018.00173

**Published:** 2018-12-18

**Authors:** Roger J. Morris

**Affiliations:** Department of Chemistry, King’s College London, London, United Kingdom

**Keywords:** membrane protein, GPI (glycosylphosphatidylinositol), adhesion, signaling system, mutant mice, neuron, astrocyte

## Abstract

Thy-1 is possibly the smallest of cell surface proteins – 110 amino acids folded into an Immunoglobulin variable domain, tethered to the outer leaflet of the cell surface membrane via just the two saturated fatty acids of its glycosylphosphatidylinositol (GPI) anchor. Yet Thy-1 is emerging as a key regulator of differentiation in cells of endodermal, mesodermal, and ectodermal origin, acting as both a ligand (for certain integrins and other receptors), and as a receptor, able to modulate signaling and hence differentiation in the Thy-1-expressing cell. This is an extraordinary diversity of molecular pathways to be controlled by a molecule that does not even cross the cell membrane. Here I review aspects of the cell biology of Thy-1, and studies of its role as deduced from gene knock-out studies, that suggest how this protein can participate in so many different signaling-related functions. While mechanisms differ in molecular detail, it appears overall that Thy-1 dampens down signaling to control function.

## Introduction

When I last reviewed Thy-1 (Morris, 1992), we knew a lot about its chemical structure and its expression in neuronal and lymphoid tissues, and were just starting to glimpse its function (Tiveron et al., 1992). I titled that review “Thy-1, the Enigmatic Extrovert on the Neuronal Surface.” Enigmatic, because whenever anyone looked carefully at Thy-1, they invariably found it awkward, refusing to do the expected and thereby revealing new mechanisms. And extrovert, because there simply is so much Thy-1 that it cannot be ignored, an abundance that led to its being the first chemically characterized mammalian membrane protein (apart from red blood cell proteins) (Williams et al., 1977), and a feature that surely is central to understanding its role.

Since then, neuronally expressed Thy-1 has been demonstrated to be an adhesive ligand for the integrin α_v_β_3_ (Leyton et al., 2001; Hermosilla et al., 2008), inhibiting neurite extension from Thy-1 expressing neural cells and promoting focal adhesion formation, cell motility and inflammatory activation on the integrin-expressing partner, mature astrocytes (Hermosilla et al., 2008; Avalos et al., 2009; Herrera-Molina et al., 2012; Kong et al., 2013; Lagos-Cabre et al., 2017). Subsequently, additional integrins α_v_β_5_ (Zhou et al., 2010), α_5_β_1_ (Fiore et al., 2014), α*_C_*β_2_ (Choi et al., 2005), and α*_M_*β_2_ (Wetzel et al., 2004) have been shown to be Thy-1 receptors; syndecan-4, thrombospondin and sulphated glycans are frequently co-receptors in focal adhesions (Hueber et al., 1992; Rege et al., 2006; Avalos et al., 2009; Kong et al., 2013; Fiore et al., 2014); and CD97, a 7-transmembrane G-protein coupled adhesive receptor, has also been shown to be a Thy-1 receptor (Wandel et al., 2012), expanding the families of known Thy-1 receptors.

Each of these receptors activates Thy-1 to function as a differentiation-triggered switch in different tissues to control an increasingly diverse set of signaling pathways, either directly [e.g., Cbp/Csk/Src-family kinases (Chen et al., 2009c; Maldonado et al., 2017), Fas (Cohen et al., 2009; Liu et al., 2017), PPARγ (Varisco et al., 2012), and the binding and uptake of mesenchymal stem cell derived extracellular vesicles (Shentu et al., 2017)]. Interdependence of Thy-1 with Wnt/β-catenin expression is emerging as an important regulator of bone and liver development (Cheng et al., 2014; Picke et al., 2018). Inappropriate expression of Thy-1 in these tissues affects oncogenesis, often (but not always) acting as a tumor suppressor (Kumar et al., 2016). Thy-1 usually acts in *trans*, binding to a receptor present on another cell and thereby regulating signaling in both cells (Herrera-Molina et al., 2013) but on a subset of lung fibroblasts it acts in *cis*, binding to the ‘inactive’ (bent) conformation of α_v_β_3_ integrin on its own surface to act as a mechanosensitive detector (Fiore et al., 2015).

An important feature of activation of Thy-1 via its physiological receptors is that they act monovalently (e.g., soluble, monovalent α_v_β_3_-Fc substitutes for the astrocytic receptor, inhibiting neurite outgrowth by neurons); cross-linking by a divalent receptor is not necessary (Herrera-Molina et al., 2012; Fiore et al., 2014; Maldonado et al., 2017). When protein A (PA) has been used to cross-link the Fc regions to produce divalent (α_v_β_3_-Fc)_2_-PA, the response (neurite retraction; size of Thy-1 clusters) increased by ∼50% (Herrera-Molina et al., 2012) but this is be expected given that divalency squares the effective affinity (‘avidity’) of a membrane-bound ligand (Morris, 1994) and cross-linking would necessarily combine separate small clusters into bigger ones.

So, is Thy-1 still enigmatic? Very much so! That such a small protein can contribute to the fine control of such diverse cellular interactions is remarkable. Of course, now that we know that mankind has at most 22,000 genes (Abascal et al., 2018), just a few times more than the simplest bacterium, it follows that most mammalian proteins must be able to combine with other macromolecules (lipids, carbohydrates and nucleotides, as well as other proteins) to produce by combinatorial diversity the vast range of specialist structures and functions needed to create our extraordinarily complex bodies and minds. Current interest is focused on the ability of Intrinsically Disordered Proteins to adopt multiple conformations that allow them to form different functional complexes with different partners (e.g., Fu and Vendruscolo, 2015; Uversky, 2017; Berlow et al., 2018). As a single immunoglobulin domain stabilized by two internal disulphide bonds (Williams et al., 1977), Thy-1 is anything but intrinsically disordered. But it is able to influence, and be influenced by, its lipid environment, as I will argue here. Thy-1 is a prime exemplar of a membrane organizer for this post-genomic era: one little protein, modified with lipids and carbohydrates, that combines with multiple receptors and signaling pathways to fine tune diverse physiological functions.

## Thy-1 Abundance – Why So Much?

Arguably the most detailed determination of the abundance of cell surface molecules for nucleated cells is for rat lymphocytes (Barclay et al., 1993). There are 10^6^ molecules of Thy-1 per cell on rat thymocytes, amounting (in molar terms) to around 80% of the total cell surface protein, covering somewhere between 5 and 25% of the thymocyte surface at an average density of 7,100 molecules per μm^2^. The next most abundant thymocyte protein is the large adhesive sialoglycoprotein CD43 (10^5^ per thymocyte) (Barclay et al., 1993). The core signaling unit, the T Cell Receptor/CD3 complex, occurs at around 10^4^ molecules per cell (Valitutti et al., 1995), as do co-receptor molecules CD4 and CD8 (Barclay et al., 1993). Thus, on thymocytes, Thy-1 is 100 times more abundant than mainstream signaling proteins, and 10x more abundant than a major adhesion protein. In the nervous system, the level of Thy-1 on axons is somewhat lower (500–1,500 molecules per μm^2^; Beech et al., 1983) but this is still much higher than typical levels of expression on cell lines (e.g., 20 molecules of Thy-1 label per μm^2^ on RBL-2H3 mast cells; Veatch et al., 2012). If Thy-1 is expressed in excess of any signaling or adhesive need, are there additional beneficial effects, for instance conferred by Thy-1’s organization of its immediate lipid environment, that explain its high abundance?

## Distribution of Thy-1 on Naked Neuronal Membrane

**FIGURE 1:**
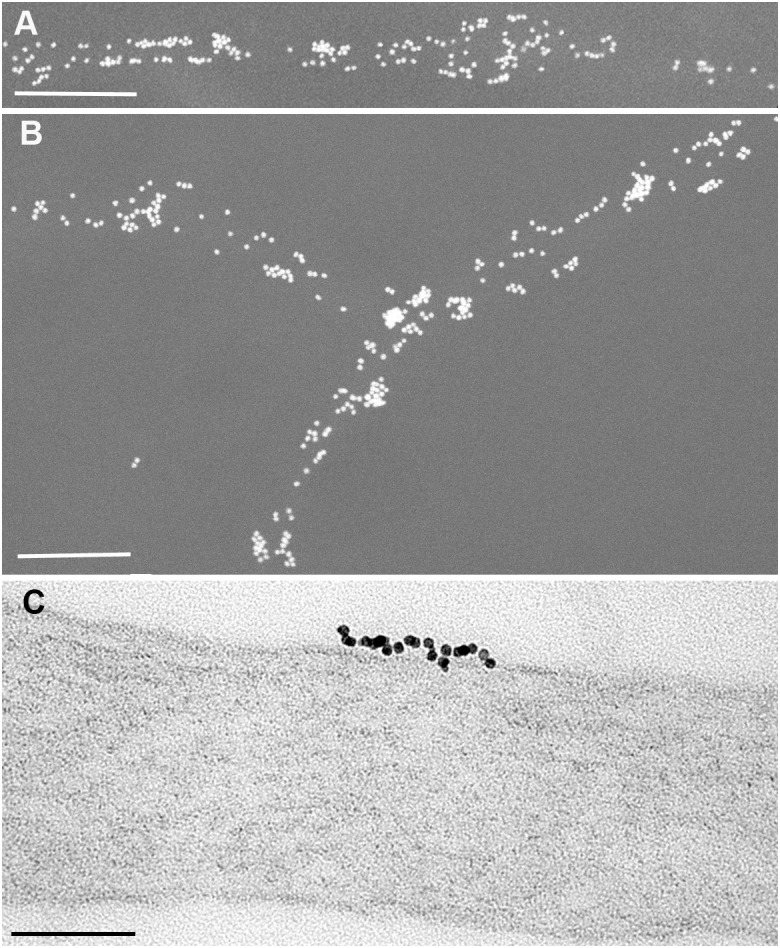
Immunogold labeling of Thy-1 on axons of adult sensory neurons growing in culture, seen **(A,B)** as 40 nm Fab-gold viewed by electron backscattering in an SEM; and **(C)**, 5 nm Fab-gold seen in the TEM. In **(A,B)**, the culture was fixed for 20 min at 4°C with 0.5% paraformaldehyde + 0.5% glutaraldehyde, before washing and labeling with OX7 Fab coupled to 40 nm gold. In **(C)**, a live culture was labeled for 30 min at 4°C with monovalent 5 nm OX7 Fab gold before fixation as above and processing for TEM. Scale bars are 1 μm in **(A,B)** and 100 nm in **(C)**. From Madore et al. (1999) and Morris et al. (2004).

What does this abundant surface expression look like? Figures 1A,B show Thy-1 immunolabelling (Fab OX7 antibody fragments coupled directly to 40 nm gold) on axons growing in tissue culture from adult sensory neurons, viewed in a scanning electron microscope (SEM) by electron backscattering in which the gold appears as white spheres and the axons are evident only by their gold label (Madore et al., 1999). The 40 nm gold is huge compared to Thy-1, which has a 6 nm Stokes diameter (Barclay et al., 1975). Several Thy-1 molecules could underlie each gold particle. No detergent, necessary to expose Thy-1 in adhesion complexes to Fab antibodies, has been added, so this is a view only of the naked upper and lateral surfaces of the axons.

By any definition, this is clustered distribution of gold label: some axonal surface is heavily labeled, and some not at all. Clustering of surface molecules into nm domains is often the result of receptor activation. Could that be the case with Thy-1 on axons? For instance, could Thy-1 on the upper (non-adhesive) axonal surface already be bound in *cis* to receptors on that surface, as it does with α_v_β_3_ to act as a mechanosensor on lung fibroblasts (Fiore et al., 2015)? Sensory neurons express a range of β1 integrins including α5β1 (Tomaselli et al., 1993) a known Thy-1 receptor (Fiore et al., 2014), as well as Syndecan-4 (Lin et al., 2015). Both are components of focal adhesions, and may affect Thy-1 distribution on the lower surface of the axons that adhere, in these cultures, to a laminin substrate. The *cis*-interaction of Thy-1 with α_v_β_3_ appears to be specific to mechanosensing in the lung, and has been specifically excluded on neuronal cells (Maldonado et al., 2017). As far as we know, Figure 1 shows examples of the distribution of unactivated Thy-1 on naked neuronal surface.

Figure 1C shows a higher power view of a large (23 gold labels) Thy-1 cluster seen in the Transmission Electron Microscope (TEM) of a section of axon labeled with monovalent Ox7 Fab-5nm gold, a label that is slightly smaller than Thy-1 and so able to label each Thy-1 molecule. Thy-1 is packed very tightly, with little if any room for additional interposed surface protein. This particular labeled patch is 125 nm in length, rather larger than the 10–20 nm range often identified as the size of membrane rafts of GPI-APs (Garcia-Parajo et al., 2014).

How reliable is our interpretation that the clustered gold label is an accurate reflection of the distribution of Thy-1 on the membrane? (Veatch et al., 2012) immunogold labeled unactivated IgE bound to FcεRI receptors on unstimulated RBL-2H3 mast cells, and obtained a distribution of 1-10 gold particles per patch. Extensive mathematical analysis and control experiments showed that their gold labeling was “dominated by multiple gold particles binding to single target proteins.” Only when the IgE was activated, thereby activating its FcεRI receptor, did true receptor clustering occur. For their study, they used commercial divalent IgG antibodies raised against divalent Ig of other species. In my experience, one generally gets at least six molecules of secondary antibody binding to dimeric IgG primary antibody (that is, at least three epitopes on the 75 kDa monomer unit of IgG are recognized by anti-IgG antibody). The Stokes’ diameters of IgG and IgE are 10.4 and 12.0 nm respectively (Griffiths and Gleich, 1972), providing enough distance from the Fc𝜖RI receptor to allow multiple 10 nm gold labels to bind, as found in this study. In contrast, we make our own anti-GPI-AP antibodies in house, digest them to monovalent Fab fragments which we directly couple to gold. Since 5 nm gold particles have a surface area of 78.6 nm^2^, Fab (Stokes diameter 6.3 nm; Griffiths and Gleich, 1972) conjugates multiply to each 5 nm gold particle which thereby becomes multivalent. When we couple to gold we dilute the immune Fab with an excess of non-immune Fab until 30–50% of the gold does not bind at all to antigen and so contains no immune Fab, leaving the residual 50–70% predominantly labeled with 1 immune Fab to produce monovalent gold (see Supplementary Material, Sunyach et al., 2003). For Thy-1, we use monoclonal antibody OX7 directed to a single epitope centered on Arg 89 (Mason and Williams, 1980). Monovalent binding of gold to Thy-1 is built into our reagents. In addition to the live cell labeling of Figure 1C, to preclude any possible probe-induced movement of Thy-1 (or PrP^C^), we also pre-fixed cells with fixative containing 0.5% EM grade glutaraldehyde, which covalently fixes proteins within msec, unlike paraformaldehyde that initially forms labile bonds that take several hours to covert to stable covalent bonds (Morris and Barber, 1983) allowing considerable post-fixed movement of GPI-AP (Mayor et al., 1994). The distribution of label we find with live or fixed tissue is indistinguishable by eye, and reflects the trouble we have taken to identify fixation conditions that immobilize each GPI-AP without destroying their epitopes recognized by our antibodies (Morris and Barber, 1983; Ford et al., 2002).

**FIGURE 2:**
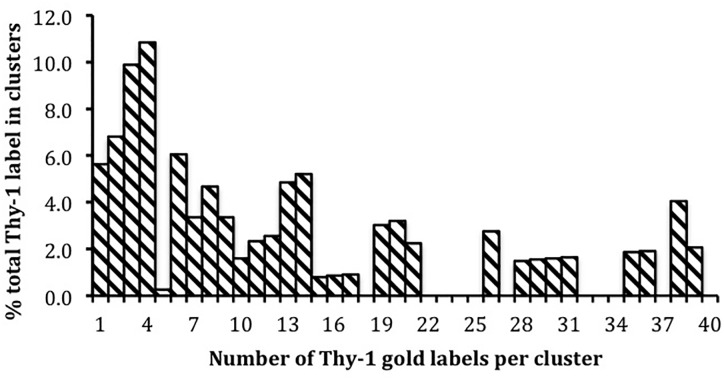
Distribution of monovalent Thy-1 Fab-gold (10 nm) label in clusters containing different numbers of gold particles on the surface of cultured adult sensory neurons. Data from Brügger et al. (2004); 1881 gold particles in 387 clusters were counted.

We estimated the diversity of size of Thy-1 clusters on cultured adult neurons in (Brügger et al., 2004) taking as the definition of a cluster, gold particles that were within 20 nm of each other. I have re-plotted that data in Figure 2 to show the size distribution: 5.8% of label was solitary, a third in clusters of fewer than 5, and 50% of all label occurred in clusters of 8 or fewer. However, larger clusters were also observed. (Suzuki et al., 2012) note that the number of GPI-AP CD59 molecules per cluster, prior to ligand binding, rises to include clusters of > 10 CD59 when the level of surface expression is increased from 0.16 to 0.90 copies per μm, still three orders of magnitude below Thy-1’s level on neurons. We used a crude definition of ‘cluster’ since our interest was not in the size of clustered Thy-1, but in whether it and PrP^C^, a GPI-AP expressed on the same neuronal surface, just nm away from Thy-1 label, occupy the same, or identifiably different, membrane ‘rafts.’ At that time, membrane ‘rafts’ were treated as a single specialized lipid environment in which co-existed all GPI-APs expressed by the cell. Our demonstration (Brügger et al., 2004) that Thy-1 and PrP membrane domains could be isolated separately and had, reproducibly, different lipid compositions, was I believe the first indication that GPI-AP’s on the same membrane tailored their lipids to suit their individual requirements, a point since made independently (Surviladze et al., 2007).

I suspect the greatest difference between our, and others’, studies on GPI-AP is that we study endogenously expressed Thy-1 on primary cultures of mature neurons, having first acquired a detailed knowledge of the *in vivo* expression of the molecules (Morris, 1992; Ford et al., 2002). Standard cell lines are seductively convenient, but their ease of transfection compared to differentiated primary cells highlights their more porous membrane. Neuronal function is completely dependent upon the non-permeability of their surface membrane; and because neurons must sustain stability of their synaptic networks over our life-times, they are post-mitotic, very different from rapidly dividing cell lines, with vastly more intricate and longer-lived specialization of their cell surface. I question the reliance of so much work on cell lines to study ‘rafts’ as key organizers of cell surface function, when the cell lines have been selected for decades on the permeability of their membranes to transfection, and their high rate of uncontrolled cell division.

## Distribution of Thy-1 on Neuronal Membrane Adhering to Astrocytes

This topic is covered authoritatively elsewhere (Herrera-Molina et al., 2012; Maldonado et al., 2017). I draw your attention here to another recent study (Nemoto et al., 2017) because of the promise it shows in applying single molecule tracking to follow GPI-APs on the adherent surface of differentiating neurons in cultures. The cultures were of neonatal rat hippocampal neurons growing on astrocytes, at two points of postnatal differentiation (1 and 2 weeks in culture, when dendrites and axons are growing and synaptic networks start to form), observed at 37°C by TIRF microscopy focused on the substrate-adhesive surface membrane in contact with the underlying astrocytes (Nemoto et al., 2017). Individual molecules of PrP^C^ and Thy-1 exhibited identical overall behavior – long (seconds) periods of immobility interspersed with rapid diffusion. Such stalled periods are typical of GPI-APs that have formed a signaling complex that temporarily anchors the GPI-AP to the cytoskeleton (Suzuki et al., 2007a,b). The cell line CHO-K1, not of neuronal origin and not natively expressing Thy-1 (Suzuki et al., 2012) was used as a control.

The specific behavior of Thy-1 and PrP^C^ differed: for instance, around 50% of Thy-1 was immobilized at any time, compared to 71% of PrP^C^; and the duration of Thy-1 mobile phases lasted more than twice that of PrP^C^ ((Nemoto et al., 2017), reproduced in Table 1). The tantalizing result in this study, however, was the duration of immobilized phases for both PrP^C^ and Thy-1, which showed exceptionally large standard deviations. For Thy-1, both means and standard deviations increased markedly during the 2nd week of growth; and for PrP^C^, decreased. Technical problems limit the accuracy of the Thy-1 results (Nemoto et al., 2017) but Thy-1’s rapidly increasing immobilization during neuronal maturation would be expected if it is binding to a variable but rising expression of its astrocytic α_v_β_3_ receptor (Leyton et al., 2001; Herrera-Molina et al., 2012) which would immobilize Thy-1 on the adhesive substrate surface. The opposite, rapidly shortening immobilization of PrP^C^ could be due to an increasing expression of its endocytic partner, LRP1 (Parkyn et al., 2008) that rapidly recycles PrP^C^ on neurons every few minutes, leading it out of rafts and into coated pits, from where it is endocytosed then sorted in recycling endosomes and returned to neuronal surface rafts (Sunyach et al., 2003).

**Table 1:** Duration (photobleaching corrected) of MOBILE and IMMOBILE states, and % protein Immobilized at any time (in seconds), for Thy-1 and PrP^C^ on cultured hippocampal neurons at 37°C.

		Mobile Duration mean ± sem (s)	Immobile Duration mean ± sem, (s)	% Immobile GPI-AP mean ± sem
Neurons, 1 week	Thy-1	0.79 ± 0.21	4.11 ± 4.29	48.5 ± 8.2
	PrP	0.41 ± 0.049	4.69 ± 2.20	71.1 ± 3.4
Neurons, 2 weeks	Thy-1	0.99 ± 0.19	10.41 ± 15.02	54.8 ± 4.4
	PrP	0.38 ± 0.074	2.75 ± 1.43	71.8 ± 5.6
CHO-K1 cell line	Thy-1	1.91 ± 0.41	1.84 ± 0.65	51.7 ± 4.5
	PrP	0.75 ± 0.091	1.74 ± 0.36	71.4 ± 2.8


*Data taken from Nemoto et al. (2017).*

The application of single molecule, as well as super-resolution, fluorescent techniques to study Thy-1’s action on the surface of cells that are interacting with their neighbors in complex primary cultures promises to add much to our understanding of molecular mechanisms of membrane function.

## The Stress Imposed by Thy-1 and Other GPI-APs on Their Membrane Lipids

When Thy-1, embedded in a membrane, has its GPI-anchor cleaved by either phospholipase C or D, allowing the protein-glycosyl component to float free of the membrane, a large conformational change occurs on the opposite face of Thy-1, where Arg 89, that specifies the Thy-1.1 allele recognized by the OX7 monoclonal antibody, resides (Barboni et al., 1995). Despite OX7 being a particularly high affinity antibody (Mason and Williams, 1980), PLC/D cleavage of Thy-1 caused pre-bound OX7 to be released, and prevented any more antibody binding to its site. The conformational shift was evident in the Circular Dichroism spectrum of human Thy-1; and by Molecular Dynamics modeling of the effect of deacylation upon Thy-1 (Barboni et al., 1995). Similar conformational changes accompany deacylation of other membrane-bound GPI-APs (Butikofer et al., 2001; Paulick and Bertozzi, 2008; Bradley et al., 2013) suggesting the conformational effect of the membrane on the lipid-anchored protein is a general property of GPI-APs.

If the membrane can exert such strong conformational restraint upon Thy-1, then Newton’s Third Law requires that the fully acylated protein exert an equal and opposite force upon the membrane. Is this an inconsequential curiosity, or is it telling us something functionally important about the interaction between GPI-AP’s and their local membrane environment? While the raft membrane around GPI-APs is generally agreed to be in an ordered phase, the multiplicity of lipid species present in membranes allows multiple ordered phases to be formed (Brügger et al., 2004; Surviladze et al., 2007). There is no single lipid environment in ‘rafts’ – each protein tailors its lipids to suit its specific needs. Further, there is no single GPI-anchor – post-translational modification of the anchor is also tailored to the needs of individual proteins (Fujita and Kinoshita, 2012; Puig et al., 2014). And the phase of raft lipids is determined, not just by the ordered lipids, but also by the disordered, polyunsaturated lipids surrounding them (Bakht et al., 2007). Could the effect of the GPI-AP tension on its immediate ‘raft’ environment be unique to each GPI-AP and its expressing cell, and determine not only the conformation of the protein, but also the distinctive composition and phase properties of its surrounding lipids?

Any contribution to membrane tension conferred by GPI-APs will be altered by ligand binding, which in Thy-1’s case on neurons may differ from general expectations (Garcia-Parajo et al., 2014). In the larger clusters (e.g., Figure 1C), Thy-1 molecules are closely adjacent, about 6 nm apart, each occupying ∼28 nm^2^ (similar to the estimate of Suzuki et al., 2012). Integrins in focal adhesions occupy ∼100 nm^2^ (Brinkerhoff and Linderman, 2005), giving an equivalent diameter of ∼11.2 nm. On astrocytes, the spatial distribution of α_v_b_3_ integrins will presumably force bound Thy-1 to spread out to fourfold lower density than in a non-activated cluster.

Further, ordered domains formed in the outer leaflet induce complementary ordered domains in the apposed inner leaflet (Allender and Schick, 2006; Collins and Keller, 2008; Kiessling et al., 2009; Raghupathy et al., 2015). Should each GPI-AP imprint its distinctive presence on its outer leaflet lipids, do they in turn pass that imprint on, to selectively recruit specific inner leaflet lipids to their raft? The interactive composition of the ordered domain in both leaflets could determine which of available transmembrane adaptor proteins (e.g., LAT, PAG/Cbp, NTAL and LIME; Horejsi et al., 2004) dock with Thy-1. These adaptor proteins have minimal (a few amino acids) extracellular domains, and functionally require palmitoylation of Cys residues located at the cytoplasmic end of their transmembrane domain that interact with the inner leaflet of raft membrane (Tanimura et al., 2006). Inner, as well as outer, lipid leaflets of a raft are involved in establishing the appropriate environment for interaction with signaling complexes.

Since the outer leaflet influences the conformation of GPI-APs, does the inner leaflet similarly impose conformational constraints upon cytoplasmic diacylated proteins such as the Src Family Kinases (SFK)? Cytoplasmic diacylation attaches C14/C16 or C16/C16 saturated lipids directly to the N-terminal amino acids, with no glycosyl linker (van’t Hof and Resh, 2000). A membrane effect upon protein conformation could be even stronger for these inner leaflet proteins. Does protein/lipid tension operate on both sides of the surface membrane?

Interest in membrane tension has primarily focused upon large scale parameters such as membrane curvature and line tension (e.g., Huttner and Zimmerberg, 2001; Kuzmin et al., 2005), but is now, in studying the mechanism of signaling by the T Cell Receptor (TCR), moving to direct mechanosensor effects on protein conformation that allow ‘catch’ and ‘slip’ bonds to differentiate between activation by foreign, or self, antigen (Chakraborty and Weiss, 2014; Liu et al., 2014; Sibener et al., 2018). The precise (Å scale) positioning of different proteins of the signaling complex is also emerging as a key differentiator of the course of TCR signaling (Chakraborty and Weiss, 2014). A similar tension-dependent catch-and-slip mechanism has been found for the co-operative binding of Thy-1 by α_5_β_1_ integrin and Syndecan-4 in contractility-dependent mechanosignalling of melanoma cells (Fiore et al., 2014). Could tension between membrane proteins and lipids be a factor here?

These speculative questions reflect my view that we have over-simplified our analysis of membrane lipids and proteins; we need better assay systems and finer grain analysis to fully understand membrane mechanisms. Membrane lipids are generally considered in overall classes (e.g., saturated vs. unsaturated; glycerolipids vs. sphingolipids), and similar members of the same class can undoubtedly substitute for each other in some aspects of membrane function. However, it is now clear that highly specified individual membrane lipids (e.g., C18:0/20:4 phosphatidylserine, and not other phosphatidylserines) are critical for individual steps in cytokinesis (Atilla-Gokcumen et al., 2014). More generally, individual lipids are being shown to be required at different stages in a cell’s biology (Storck et al., 2018). Specific lipids in both leaflets of the membrane marshaled by individual GPI-APs could be functionally important in the transfer of information across the surface membrane. While the variety of receptors with which Thy-1 interacts is controlled by binding sites on Thy-1’s protein domain, the precise lipids that surround Thy-1, which could differ in different cells and states of differentiation, could help select the transmembrane signaling mechanism that is addressed.

## Isolation of Lipid ‘RAFTS’ as Detergent Resistant Membrane (DRM)

It clearly would be a huge advance if individual rafts could be isolated and their full range of components identified. Endless experiments have sought to do just this, particularly using the resistance to solubilization by mild detergents of cholesterol-condensed saturated lipids to isolate rafts as DRM.

Significant opinion holds that rafts cannot be isolated – they are transient, critically temperature dependent (Honerkamp-Smith et al., 2008) assemblies whose defining characteristics are destroyed in the instant they are exposed to detergent (e.g., Munro, 2003). This view arises from the repeated demonstration by single molecule tracking that individual raft molecules are highly mobile; and even when immobilized as part of a signaling complex, they remain so for seconds at most (e.g., Shibata et al., 2012; Nemoto et al., 2017; Suzuki et al., 2007a,b, 2012, 2018). However rapid the motility of individual components, the individual raft can be considerably longer lived; for instance, integrins continuously enter and leave stationary focal adhesions (Shibata et al., 2012; Tsunoyama et al., 2018). Fast turnover of individual components allows continuous fine tuning of the strength and duration of signaling, that can persist for minutes while being modified on a second by second basis (Suzuki et al., 2007a; Tian et al., 2007; Harding and Hancock, 2008).

An alternative case for dismissing DRMs comes from studies of the mechanism of detergent solubilization of model bilayers, where the detergent induces the artefactual formation of subdomains in the membrane (Heerklotz, 2002; Heerklotz et al., 2003; Lichtenberg et al., 2005, 2013). However, models are proving poor substitutes for real cell membranes (Lee et al., 2015). More tellingly from a physicist’s viewpoint, since the classical Lo phase is remarkably temperature stable (Ipsen et al., 1987) as are the surface membranes of cells (Lee et al., 2015), it must be possible to isolate DRMs at physiological temperature if they truly are derived from membrane rafts.

Using the standard method for DRM isolation, we and others have shown that the DRMs obtained are fusions of totally different membranes that do not preserve the outer/inner leaflet distinction of *in vivo* membranes, and indeed contain very few inner leaflet lipids; they are several μm in size, orders of magnitude greater than any raft is thought to be (Madore et al., 1999; Morris, 2010).

We also, using appropriate solubilization conditions, have immunoaffinity isolated separately Thy-1 and PrP^C^ from brain membrane DRMs (Madore et al., 1999; Brügger et al., 2004). Each DRM had, very reproducibly, distinctly different lipid compositions (Brügger et al., 2004). Similarly Thy-1 and Fc𝜖RI, again located in near-adjacent but separate clusters on the mast cell surface as seen by SEM (Veatch et al., 2012) have been immuno-isolated as separate DRMs, again with different lipid compositions (Surviladze et al., 2007). And, most remarkable of all, (Han et al., 2009) used bulk-isolated DRMs from Fc𝜖RI-activated mast cells to follow the temporal sequence of activation from ligand binding to receptor engagement with the actin cytoskeleton.

We reasoned that the critical difference in the methods used lies in the ions present, including Ca^2+^ released by ruptured cells. Not only are bilayer lipids asymmetrically distributed (Morris, 2010); the cations Ca^2+^ and Na^+^ bathe the outer leaflet, Mg^2+^ and K^+^ bathe the inner leaflet. These monovalent cations contribute significantly to the bilayer strength of individual lipids (Garcia-Manyes et al., 2005, 2010; Beedle et al., 2015). The most critical effect is the release of Ca^2+^ from solubilized cells upon the matrix of Phosphatidyl Serine (PS^-^) interleaved with Phosphatidyl Ethanolamine (PE) to form the inner leaflet. Ca^2+^, gaining access to the inner leaflet, chelates the negatively charged PS^-^, thereby withdrawing it from the PE lattice, which thus destabilized (Cullis and Verkleij, 1979; Tilcock and Cullis, 1981; Tokutomi et al., 1981; Bally et al., 1983) would be quickly removed by the detergent leaving monolayer fragments of outer membranes that fuse, thus producing the artifacts mentioned above (Morris, 2010).

We therefore used for solubilization an ‘intracellular’ buffer containing EGTA to mop up Ca^2+^ as it is released from cells; and used concentrations of K^+^, Mg^2+^ and acetate to mimic the intracellular environment. We could isolate DRMs at 37°C (and even at 55°C) that were a distinct improvement over isolation with other buffers at 4°C. Specifically, the 37°C DRMs had a more balanced proportion of inner and outer membrane lipids; Thy-1 and PrP^C^ were isolated completely separately; intracellular ‘raft’ components, such as Fyn and Flotillin were more completely recovered in the isolated DRMs; and actin (which we have never previously seen in any DRM), was isolated with Thy-1 but not PrP^C^ DRMs (Chen et al., 2009a,b). Src kinase, of particular relevance to Thy-1 signaling (Chen et al., 2009c; Maldonado et al., 2017) is untypical within the Src family of kinases (SFK) in being anchored to the inner membrane surface, not by two diacyl chains, but by a myristate plus basic protein patch (van’t Hof and Resh, 2000). The positively charged patch binds to negatively charged phosphatidylinositol patches on the inner surface membrane, linking the kinase to the membrane. Only when using the intracellular buffer have we seen substantial Src co-isolate with DRMs in the light fractions of a sucrose gradient at any temperature, much less at 37°C (Chen et al., 2009b).

In these studies we compared the benchmark DRM detergent, Triton X100, with Brij 96, which differ in the hydrophobic tail of Triton X-100 being short, disrupting primarily the interfacial region and outer leaflet, whereas Brij 96 has a longer, bilayer-spanning hydrophobic tail. Overall, Brij 96 gave a better yield of proteins and lipids in the DRM fractions, but these were marginal compared to the use of intracellular buffer (Chen et al., 2009a,b).

**FIGURE 3:**
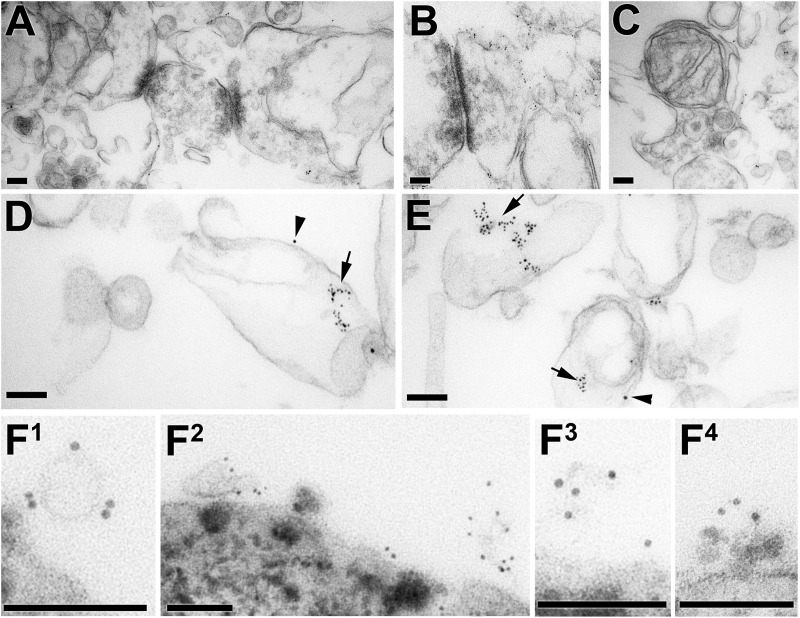
TEM photographs documenting progress in isolating Thy-1 and PrP^C^ in membrane fragments of a size and composition expected of ‘rafts,’ after solubilization of brain membranes for 15 min at 37°C in Brij 96 with intracellular buffer (Chen et al., 2009b; Morris et al., 2011). **(A–E)** Show samples recovered from a sucrose gradient; **(F^1^–F^4^)** are samples recovered immediately after solubilization (without the density gradient step) by their binding to anti-Thy-1 magnetic beads. Scale bars throughout are 100 nm. **(A–C)** Are detergent-resistant membranes recovered in the high density sucrose at the bottom of the gradient, with synaptic junctions recognizable in **(A,B)** by the post-synaptic densities; some pre-synaptic vesicles also remain. A remarkably intact mitochondrion in seen in **(C)**. **(D,E)** Show DRMs isolated from the light sucrose density fraction of the same gradient after centrifugation at 200,000 *g* for 18 h, immunolabelled for PrP^C^ (5 nm gold, arrows) and Thy-1 (10 nm gold, arrowheads). The DRMs are near μm in size, and the immunolabelled areas are a very small fraction of the whole vesicle. **(F^1^–F^4^)** Show brain membranes solubilized in Brij 96 in intracellular buffer at 37°C, with magnetic beads labeled with OX7 anti-Thy-1 IgG added after 5 min. At 15 min the beads were collected magnetically, washed and incubated with 5 nm gold labeled with OX7 Fab. The large heterogeneous objects at the bottom of **(F1–F^4^)** are sections of magnetic beads.

Some salient features of detergent solubilization of brain membranes, using 0.5% Brij 96 at 37°C with the intracellular buffer, are shown in Figure 3 (Morris et al., 2011). The top panel (Figures 3A–C) shows membranes that remain in the bottom (highest density) fraction after sucrose gradient fractionation. Remarkably, synaptic junctions with clearly identifiable pre- and post-synaptic components, and mitochondrial inner membrane, are preserved in this buffer. Although not sufficiently lipid-rich/protein-sparse to float in the low density fractions on the gradient, major functional elements of brain retain recognizable structure under these solubilization conditions, indicating that detergent resistant membrane is a much more inclusive category than just low density DRMs. It is the low protein:lipid ratio of DRMs that makes them float with the lipids, rather than be retained in the high density fractions of the gradient.

Figures 3D,E show examples of DRMs isolated at low density after overnight centrifugation on the sucrose gradient. Many of the vesicles remain large, and only small sectors of them label for either Thy-1 or PrP^C^ (Chen et al., 2009b). This suggests that membrane fusion of Thy-1 and PrP^C^ DRMs with other DRMs containing neither of these two GPI-APs was occurring, either during solubilization or during the 18 h ultracentrifugation step that specifically concentrates the light DRMs.

We therefore reduced the entire procedure to just 15 min – 5 min solubilization followed by 10 min immunoaffinity isolation on magnetic beads, all at 37°C, which yielded 20–50 nm membrane fragments bound to the magnetic beads, finally of the size expected of membrane ‘rafts’ (Figures 3F^1^–F^4^).

Circumstances forced me to close down my lab shortly after this paper was submitted, so I have not progressed this research. But for anyone with an in-house supply of relevant antibodies, I suggest adapting this approach to your material is likely to give you access to raft membrane.

## Analysis of Thy-1^-/-^ (Knockout) Mice: the Function of Thy-1 *In Vivo*

We produced and analyzed Thy-1^-/-^ mice in the heady days of the ‘90’s when careers were launched, or aborted, on the post-doc’s ability to find an interesting behavioral phenotype in the analysis of mice in which they had inactivated (knocked out) a specific gene. Everyone was using the same embryonic stem cells derived originally from a 129/Sv/Ev strain mouse. The phenotype most commonly reported was excessive aggression.

Our mice too were remarkably aggressive. Our male littermates, if housed in the same cage, killed each other. I placed an experienced wild type (Thy-1^+/+^) male into a cage with two Thy-1^-/-^ females to initiate breeding, which usually started very quickly. Within a minute the male was clinging to the bars on the top of the cage, squealing to be let out. We outbred the mice, and within a few generations had separated the Thy-1^-/-^ gene from the aggression-inciting gene. We continued this outbreeding on the 129/Sv/Ev and C57Bl6 backgrounds; and we derived an entirely independent Thy-1 knockout in C57Bl6 embryonic stem cells, and bred both onto C57Bl6 and 129/Sv/Ev backgrounds, to obtain genetically diverse but stable strains for behavioral analysis (Mayeux-Portas et al., 2000).

I mention this because scientific standards in this burgeoning field were initially not uniformly sound. We pointed out this problem in a Letter to Trends in Neuroscience (Morris and Nosten-Bertrand, 1996), which elicited a phone call from a Nature editor lamenting my failure to send it to them, as they strongly suspected there was an underlying problem with these recurrent findings of aggression. An interesting Banbury Conference on Genetic Background in Mice in 1997 laid down standards in the field (Conference, 1997). You should not believe everything you read about knockout mice of the era; but our mice provide a sound platform for analysis, and have been used in some impressive recent studies (Zhou et al., 2004; Varisco et al., 2012; Cohen et al., 2018; Picke et al., 2018).

### Lymphoid Phenotype

The 129/Sv/Ev mice were analyzed by He’s lab studying the development of thymocytes into T cells (Hueber et al., 1997). Overall, they conclude that Thy-1 negatively regulates TCR-mediated signaling to fine-tune activation thresholds during thymocyte differentiation. In particular, transition from CD4/CD8 ‘double positive’ thymocytes to mature single positive T lymphocytes was reduced; and a significant proportion of older Thy-1^-/-^ mice (from both 129Sv/Ev and C57Bl6 backgrounds) developed T lymphomas. The latter is documented in their Supplementary Material, which also shows TEM photographs in which adhesion between thymocytes and the thymic epithelium is defective in Thy-1^-/-^ mice, evident as gaps between the cells that are in sharp contrast to the firm intercellular adhesion normally found (Hueber et al., 1997). This confirmed earlier work (He et al., 1991; Hueber et al., 1992) that Thy-1 binds thymocytes to thymic epithelia. The occurrence of T lymphomas in older mice was, I believe, the first indication that Thy-1 is a tumor suppressor.

These studies of thymocyte differentiation rely upon the *in vivo* action of Thy-1 interacting with its receptors and signaling pathways. He’s group also examined the molecular events following activation of the T-cell receptor (TCR) signaling in isolated thymocytes (Hueber et al., 1997). Isolation of thymocytes as a single cell suspension, away from their surrounding stromal cells, presumably removes integrin binding to Thy-1. They found the initial phosphorylation steps in the kinase pathways activated by TCR engagement (phosphorylation of CD3𝜖, TCRζ, and Lck; Ca^2+^ mobilization) occurred in Thy-1^-/-^ thymocytes with around twice the intensity of the Thy-1^+/+^ controls, resulting in thymocyte proliferation at twice the rate in Thy-1^-/-^ compared to Thy-1^+/+^ thymocytes. Thus Thy-1 not only lowers TCR signaling; it apparently does so in the absence of its integrin receptor, although whether to the same extent is not known.

Beissert et al. (1998) went on to study cutaneous T cell function in the Thy-1^-/-^ mice, both *in vivo* and in culture. They found lower hypersensitivity responses in the Thy-1^-/-^ mice, but otherwise relatively normal T cell function. However, the mechanism was the opposite to that of thymocytes. Isolated Thy-1^-/-^ T cells, when stimulated via CD3 antibodies, showed lower activation of SFK and reduced mobilization of intracellular Ca^2+^. The net effect of constitutive Thy-1 loss was reduced overall function, but by opposite changes in SFK phosphorylation and Ca^2+^ levels in thymocytes and their T cell progeny. The outcome of TCR activation in the two cell types is very different – progression into different differentiation pathways for thymocytes, mounting a final effector function (hypersensitivity response) for T lymphocytes. Their TCR-activated intracellular signaling pathways must diverge to produce different outcomes. Mouse T lymphocytes have only 10% the Thy-1 levels of thymocytes (Acton et al., 1974); could Thy-1’s regulation of signaling when expressed at low levels, differ from that at high levels?

### Neural Phenotype

Our extensive studies of the appearance during development of Thy-1 mRNA and protein in rodent nervous system showed that *in vivo*, Thy-1 is solely expressed on neurons, and exhibited extraordinary post-transcriptional regulation of expression, the protein being kept away from growing tips of dendrites, and excluded from axons until they had synapsed on their target neurons and stopped growing. Once expressed, Thy-1 then remained as a major component of the neuronal membrane throughout adult life, reviewed in Morris (1992). The exclusion of Thy-1 protein from growing axons, so prominent *in vivo*, is not found in primary tissue culture where expression of Thy-1 mRNA rapidly leads to expression of its protein (Morris, 1992).

We therefore looked for Thy-1-dependent function in adult brain, where, in a healthy individual, a fine balance is maintained between excitatory circuits and local inhibitory neurons that allows excitatory circuits to fire, modulated by inhibitory synapses that refine, restrict and finally return the circuit to its resting potential. Failure to terminate firing of excitatory circuits can lead to recycling pulses of excitation seen as seizures.

Electrophysiological studies with Thy-1^-/-^ mice focused on the hippocampus and its associated dentate gyrus (Nosten-Bertrand et al., 1996; Errington et al., 1997; Hollrigel et al., 1998). The synaptic response of NMDA glutamate receptors at excitatory synapses of both large projection neurons (CA1 pyramidal cells) and smaller local neurons (dentate granule cells) were identical for Thy-1^+/+^ and Thy-1^-/-^ mice, except in their ability to convert persistent (tetanic) signaling into long-term enhanced responsiveness of affected synapses, called Long Term Potentiation (LTP) (Bliss et al., 2018). LTP was induced normally in the CA1 pyramidal cells, showing that basic excitatory signaling was functioning correctly, and complex long-term adaptation also progressed normally. However, similar NMDA glutamate receptor excitation of dentate granule interneurons failed, in anesthetized mice, to induce LTP. This could be overcome by adding in the recording pipette a pharmacological antagonist (bicuculline) to the inhibitory GABA_A_ receptor. Bicuculline prevents the action of local inhibitory input on the recorded cell, and with this inhibition removed, the dentate granule cells developed LTP when appropriately stimulated at their excitatory NMDA receptors. Subsequent studies on the inhibitory input to the dentate granule cells in Thy-1^-/-^ mice showed them to be identical to those of Thy-1^+/+^ mice in most respects, except that in Thy-1^-/-^ mice, spontaneous inhibitory post-synaptic currents were stronger, especially if two action potentials arrived in quick (msec) succession (Hollrigel et al., 1998).

Thus, in the hippocampus (a good model for most of the brain), the absence of Thy-1 causes too strong a response, not in the main excitatory circuitry, but in the inhibition of these circuits by local inhibitory neurons. Thy-1’s action is pre-synaptic, down-regulating inhibitory vesicle number, size, content or release, mechanisms primarily controlled by influxes of Ca^2+^ (Sudhof, 2013) rather than kinases.

Confirmation of this mode of action in the brain could be tested once a clear behavioral phenotype became evident for Thy-1^-/-^ mice (Mayeux-Portas et al., 2000). Thy-1^-/-^ mice of both genotypes failed the test of ‘social transmission of food preference,’ yet performed as well as normal mice when they were injected with a high concentration (15 mg/kg) of a GABA_A_ antagonist (pentylenetetrazole). This dose is sufficient to take a normal mouse to the verge of developing epileptic-type seizures, yet it enabled the Thy-1^-/-^ mice to be ‘normal.’ In the absence of Thy-1, the GABA_A_ inhibitory network is overly strong, approximately twice the level of normal Thy-1^+/+^ mice.

It follows that raising Thy-1 levels should weaken inhibition and make mice more prone to seizure. As it happened, we had derived a mouse line that over-expressed Thy-1, and used them in this study for transgenic rescue of the Thy-1^-/-^ mice (Mayeux-Portas et al., 2000) until a sustained fire alarm went off and a loud bell in the animal house sounded the evacuation for about 30 min. When we returned, all the Thy-1 over-expressing mice were dead, apparently from audiogenic seizure. This was not an experiment we would choose to do, but circumstances intervened to do so.

## On the Social Life of Mice....

To find a behavioral phenotype exhibited by Thy-1^-/-^ mice, we looked for normal mouse behavior whose disruption would endanger the existence of mice as a species. Olfaction is the most important sensory input for mice; olfactory memory is established via the dentate gyrus and hippocampus. We screened for olfactory memory tasks, of which the ‘social transmission of scented food’ tests a species-critical strength of murine social behavior. When a mouse leaves its burrow to forage, it will preferably eat food it knows from experience is safe; but if a new berry or seed has appeared, it may be tempted to taste it. On returning to its burrow, looking well fed and happy, the other mice gather around and smell its breath to identify what it has been eating. If they detect a new odor, and the mouse is still looking healthy, then when they go out to forage they will eat that berry or seed. Thus knowledge of what is safe to eat, and what is not, is passed around the colony. This highly evolved social behavior plays a significant role in the success of the species, and so would be maintained by evolutionary pressure. The Thy-1^-/-^ mice could be readily trained to use olfactory clues to follow a trail to food (they could smell and had olfactory-based memory); they spent as long as normal mice smelling the ‘demonstrator’ mouse when it was returned to the cage having eaten food emitting a new scent. It was only in using the information about the scented food carried on the breath of the ‘demonstrator’ mouse, to then choose which food to eat, that the Thy-1^-/-^ mice were defective. We have no reason to think this is the only phenotype displayed by these mice. Thy-1^-/-^ mice, for instance, were more flexible in searching for a new position of the hidden platform in the watermaze, which was significant in the 129/Sv/Ev but not C57Bl6 backgrounds (Mayeux-Portas et al., 2000).

## Conclusion

I started this review calling Thy-1 a pathfinder protein for the post-genomic era, because its domains (protein, lipid and two types of carbohydrate chain) confer upon it exceptional functionality that is used to regulate a diverse range of developmental decisions. In the brain, the demonstration by Leyton and colleagues of the inter-dependence of neuronal Thy-1 and astrocytic integrins is timely given the growing realization that the astrocyte is not just a general support cell, but rather is an active partner with neurons in controlling the formation and subsequent modification of synapses via contact mediated signals (Clarke and Barres, 2013). For the mature function of the nervous system, synapses must remain adaptable within relatively stable neuronal networks. I believe the effect of Thy-1 is to calm down growth signals in neurons to enable a relatively stable synaptic framework to be maintained, while promoting astrocytic growth as the brain expands during postnatal maturation leading into adulthood.

Elsewhere, Thy-1 is expressed as a molecule that decides cell fate in the development of various tissues, and limits growth to be normal, not oncogenic.

There has been excellent progress in the past decade, celebrated in this Frontiers issue, filling out these generalities with precise mechanistic details of protein-to-protein interactions enabled by heterogeneity in the membrane environment. But there remain puzzles, such as why Thy-1^-/-^ lymphocytes, isolated from their stromal cells and so from possible receptors, retain altered SFK signaling and Ca^2+^ mobilization. The stress observed exerted by the membrane on the conformation of Thy-1 is particularly intriguing, and possibly far-reaching in its implications.

In the coming decade, I think the Thy-1 membrane environment will emerge from anonymity, not just a ‘raft’ but as a highly defined, individual patch of membrane that actively enables Thy-1 to be involved in such a range of functions. In a world of Intrinsically Disordered Proteins, Individually Ordered Lipids will have their day.

## Author Contributions

The author confirms being the sole contributor of this work and has approved it for publication.

## Conflict of Interest Statement

The author declares that the research was conducted in the absence of any commercial or financial relationships that could be construed as a potential conflict of interest.

## References

[B1] Abascal F. Juan D. Jungreis I. Martinez L. Rigau M. Manuel Rodríguez J. (2018). Loose ends: almost one in five human genes still have unresolved coding status. *Nucleic Acids Res.* 46 7070–7084. 10.1093/nar/gky587 29982784 PMC6101605

[B2] Acton R. T. Morris R. J. Williams A. F. (1974). Estimation of the amount and tissue distribution of the rat Thy-1.1 antigen. *Eur. J. Immunol.* 4 598–602. 10.1002/eji.1830040904 4547712

[B3] Allender D. W. Schick M. (2006). Phase separation in bilayer lipid membranes: effects on the inner leaf due to coupling to the outer leaf. *Biophys. J.* 91 2928–2935. 10.1529/biophysj.106.086868 16877505 PMC1578461

[B4] Atilla-Gokcumen G. E. Muro E. Relat-Goberna J. Sasse S. Bedigian A. Coughlin M. L. (2014). Dividing cells regulate their lipid composition and localization. *Cell* 156 428–439. 10.1016/j.cell.2013.12.015 24462247 PMC3909459

[B5] Avalos A. M. Valdivia A. D. Munoz N. Herrera-Molina R. Tapia J. C. Chiong M. (2009). Neuronal Thy-1 induces astrocyte adhesion by engaging syndecan-4 in a cooperative interaction with alphavbeta3 integrin that activates PKCalpha and RhoA. *J. Cell Sci.* 122 3462–3471. 10.1242/jcs.034827 19723805 PMC2746130

[B6] Bakht O. Pathak P. London E. (2007). Effect of the structure of lipids favoring disordered domain formation on the stability of cholesterol-containing ordered domains (lipid rafts): identification of multiple raft-stabilization mechanisms. *Biophys. J.* 93 4307–4318. 10.1529/biophysj.107.114967 17766350 PMC2098711

[B7] Bally M. B. Tilcock C. P. Hope M. J. Cullis P. R. (1983). Polymorphism of phosphatidylethanolamine-phosphatidylserine model systems: influence of cholesterol and Mg2+ on Ca2+-triggered bilayer to hexagonal (HII) transitions. *Can. J. Biochem. Cell Biol.* 61 346–352. 10.1139/o83-048 6883167

[B8] Barboni E. Pliego Rivero B. George A. J. T. Martin S. R. Renouf D. V. Hounsell E. F. (1995). The glycophosphatidylinositol anchor affects the conformation of Thy-1 protein. *J. Cell Sci.* 108 487–497. 7539435 10.1242/jcs.108.2.487

[B9] Barclay A. N. Birkeland M. L. Brown M. H. Beyers A. D. Davis S. J. Somoza C. (1993). *The Leukocyte Antigen FactsBook.* London: Academic Press, 424.

[B10] Barclay A. N. Letarte-Muirhead M. Williams A. F. (1975). Purification of the Thy-1 molecule from rat brain. *Biochem. J.* 151 699–706. 10.1042/bj1510699 56178 PMC1172419

[B11] Beech J. N. Morris R. J. Raisman G. (1983). Density of Thy-1 on axonal membrane of different rat nerves. *J. Neurochem.* 41 411–417. 10.1111/j.1471-4159.1983.tb04757.x 6135751

[B12] Beedle A. E. Lezamiz A. Stirnemann G. Garcia-Manyes S. (2015). The mechanochemistry of copper reports on the directionality of unfolding in model cupredoxin proteins. *Nat. Commun.* 6:7894. 10.1038/ncomms8894 26235284 PMC4532836

[B13] Beissert S. He H. T. Hueber A. O. Lellouch A. C. Metze D. Mehling A. (1998). Impaired cutaneous immune responses in Thy-1-deficient mice. *J. Immunol.* 161 5296–5302. 9820502

[B14] Berlow R. B. Dyson H. J. Wright P. E. (2018). Expanding the paradigm: intrinsically disordered proteins and allosteric regulation. *J. Mol. Biol.* 430 2309–2320. 10.1016/j.jmb.2018.04.003 29634920 PMC6045455

[B15] Bliss T. V. P. Collingridge G. L. Morris R. G. M. Reymann K. G. (2018). Long-term potentiation in the hippocampus: discovery, mechanisms and function. *Neuroforum* 24 A103–A120. 10.1515/nf-2017-A059

[B16] Bradley J. E. Chan J. M. Hagood J. S. (2013). Effect of the GPI anchor of human Thy-1 on antibody recognition and function. *Lab. Invest.* 93 365–374. 10.1038/labinvest.2012.178 23358110 PMC5662197

[B17] Brinkerhoff C. J. Linderman J. J. (2005). Integrin dimerization and ligand organization: key components in integrin clustering for cell adhesion. *Tissue Eng.* 11 865–876. 10.1089/ten.2005.11.865 15998226

[B18] Brügger B. Graham C. H. Leibrecht I. Mombelli E. Jen A. Wieland F. T. (2004). The membrane domains occupied by glycosylphosphatidylinositol-anchored prion protein and Thy-1 differ in lipid composition. *J. Biol. Chem.* 279 7530–7536. 10.1074/jbc.M310207200 14660659

[B19] Butikofer P. Malherbe T. Boschung M. Roditi I. (2001). GPI-anchored proteins: now you see ‘em, now you don’t. *FASEB J.* 15 545–548. 10.1096/fj.00-0415hyp 11156970

[B20] Chakraborty A. K. Weiss A. (2014). Insights into the initiation of TCR signaling. *Nat. Immunol.* 15 798–807. 10.1038/ni.2940 25137454 PMC5226627

[B21] Chen X. Jayne Lawrence M. Barlow D. J. Morris R. J. Heenan R. K. Quinn P. J. (2009a). The structure of detergent-resistant membrane vesicles from rat brain cells. *Biochim. Biophys. Acta* 1788 477–483. 10.1016/j.bbamem.2008.11.023 19118517

[B22] Chen X. Jen A. Warley A. Lawrence M. J. Quinn P. J. Morris R. J. (2009b). Isolation at physiological temperature of detergent-resistant membranes with properties expected of lipid rafts: the influence of buffer composition. *Biochem. J.* 417 525–533. 10.1042/BJ20081385 18831713

[B23] Chen Y. Veracini L. Benistant C. Jacobson K. (2009c). The transmembrane protein CBP plays a role in transiently anchoring small clusters of Thy-1, a GPI-anchored protein, to the cytoskeleton. *J. Cell Sci.* 122 3966–3972. 10.1242/jcs.049346 19825940 PMC2773195

[B24] Cheng B. Q. Jiang Y. Zhu Q. Lin W. G. (2014). Wnt/beta-catenin aids in regulating the proliferation of hepG2 cells mediated by thy-1. *Genet. Mol. Res.* 13 5115–5127. 10.4238/2014.July.7.4 25061736

[B25] Choi J. Leyton L. Nham S. U. (2005). Characterization of alphaX I-domain binding to Thy-1. *Biochem. Biophys. Res. Commun.* 331 557–561. 10.1016/j.bbrc.2005.04.006 15850796

[B26] Clarke L. E. Barres B. A. (2013). Emerging roles of astrocytes in neural circuit development. *Nat. Rev. Neurosci.* 14 311–321. 10.1038/nrn3484 23595014 PMC4431630

[B27] Cohen P. Y. Breuer R. Wallach-Dayan S. B. (2009). Thy1 up-regulates FasL expression in lung myofibroblasts via Src family kinases. *Am. J. Respir. Cell Mol. Biol.* 40 231–238. 10.1165/rcmb.2007-0348OC 18676775

[B28] Cohen P. Y. Breuer R. Wallach-Dayan S. B. (2018). A profibrotic phenotype in naive and in fibrotic lung myofibroblasts is governed by modulations in Thy-1 expression and activation. *Mediat. Inflamm.* 2018:4638437. 10.1155/2018/4638437 30002599 PMC5996423

[B29] Collins M. D. Keller S. L. (2008). Tuning lipid mixtures to induce or suppress domain formation across leaflets of unsupported asymmetric bilayers. *Proc. Natl. Acad. Sci. U.S.A.* 105 124–128. 10.1073/pnas.0702970105 18172219 PMC2224171

[B30] Conference B. (1997). Mutant mice and neuroscience: recommendations concerning genetic background. Banbury Conference on genetic background in mice. *Neuron* 19 755–759. 10.1016/S0896-6273(00)80958-7 9354323

[B31] Cullis P. R. Verkleij A. J. (1979). Modulation of membrane structure by Ca2+ and dibucaine as detected by 31P NMR. *Biochim. Biophys. Acta* 552 546–551. 10.1016/0005-2736(79)90200-1 571738

[B32] Errington M. L. Bliss T. V. P. Morris R. J. Laroche S. Davis S. (1997). Long-term potentiation in awake mutant mice. *Nature* 387 666–667. 10.1038/42625 9192888

[B33] Fiore V. F. Ju L. Chen Y. Zhu C. Barker T. H. (2014). Dynamic catch of a Thy-1-alpha5beta1+syndecan-4 trimolecular complex. *Nat. Commun.* 5:4886. 10.1038/ncomms5886 25216363

[B34] Fiore V. F. Strane P. W. Bryksin A. V. White E. S. Hagood J. S. Barker T. H. (2015). Conformational coupling of integrin and Thy-1 regulates Fyn priming and fibroblast mechanotransduction. *J. Cell Biol.* 211 173–190. 10.1083/jcb.201505007 26459603 PMC4602038

[B35] Ford M. J. Burton L. J. Li H. Graham C. H. Frobert Y. Grassi J. (2002). A marked disparity between the expression of prion protein and its message by neurones of the CNS. *Neuroscience* 111 533–551. 10.1016/S0306-4522(01)00603-0 12031342

[B36] Fu B. Vendruscolo M. (2015). Structure and dynamics of intrinsically disordered proteins. *Adv. Exp. Med. Biol.* 870 35–48. 10.1007/978-3-319-20164-1_2 26387099

[B37] Fujita M. Kinoshita T. (2012). GPI-anchor remodeling: potential functions of GPI-anchors in intracellular trafficking and membrane dynamics. *Biochim. Biophys. Acta* 1821 1050–1058. 10.1016/j.bbalip.2012.01.004 22265715

[B38] Garcia-Manyes S. Oncins G. Sanz F. (2005). Effect of ion-binding and chemical phospholipid structure on the nanomechanics of lipid bilayers studied by force spectroscopy. *Biophys. J.* 89 1812–1826. 10.1529/biophysj.105.064030 15980180 PMC1366684

[B39] Garcia-Manyes S. Redondo-Morata L. Oncins G. Sanz F. (2010). Nanomechanics of lipid bilayers: heads or tails? *J. Am. Chem. Soc.* 132 12874–12886. 10.1021/ja1002185 20799688

[B40] Garcia-Parajo M. F. Cambi A. Torreno-Pina J. A. Thompson N. Jacobson K. (2014). Nanoclustering as a dominant feature of plasma membrane organization. *J. Cell Sci.* 127 4995–5005. 10.1242/jcs.146340 25453114 PMC4260763

[B41] Griffiths R. W. Gleich G. J. (1972). Proteolytic degradation of IgD and its relation to molecular conformation. *J. Biol. Chem.* 247 4543–4548. 4261399

[B42] Han X. Smith N. L. Sil D. Holowka D. A. McLafferty F. W. Baird B. A. (2009). IgE receptor-mediated alteration of membrane-cytoskeleton interactions revealed by mass spectrometric analysis of detergent-resistant membranes. *Biochemistry* 48 6540–6550. 10.1021/bi900181w 19496615 PMC2767325

[B43] Harding A. S. Hancock J. F. (2008). Using plasma membrane nanoclusters to build better signaling circuits. *Trends Cell Biol.* 18 364–371. 10.1016/j.tcb.2008.05.006 18620858 PMC2780343

[B44] He H. T. Naquet P. Caillol D. Pierres M. (1991). Thy-1 supports adhesion of mouse thymocytes to thymic epithelial cells through a Ca2(+)-independent mechanism. *J. Exp. Med.* 173 515–518. 10.1084/jem.173.2.515 1671083 PMC2118789

[B45] Heerklotz H. (2002). Triton promotes domain formation in lipid raft mixtures. *Biophys. J.* 83 2693–2701. 10.1016/S0006-3495(02)75278-8 12414701 PMC1302353

[B46] Heerklotz H. Szadkowska H. Anderson T. Seelig J. (2003). The sensitivity of lipid domains to small perturbations demonstrated by the effect of Triton. *J. Mol. Biol.* 329 793–799. 10.1016/S0022-2836(03)00504-7 12787678

[B47] Hermosilla T. Munoz D. Herrera-Molina R. Valdivia A. Munoz N. Nham S. U. (2008). Direct Thy-1/alphaVbeta3 integrin interaction mediates neuron to astrocyte communication. *Biochim. Biophys. Acta* 1783 1111–1120. 10.1016/j.bbamcr.2008.01.034 18346467 PMC2587321

[B48] Herrera-Molina R. Frischknecht R. Maldonado H. Seidenbecher C. I. Gundelfinger (2012). Astrocytic alphaVbeta3 integrin inhibits neurite outgrowth and promotes retraction of neuronal processes by clustering Thy-1. *PLoS One* 7:e34295. 10.1371/journal.pone.0034295 22479590 PMC3316703

[B49] Herrera-Molina R. Valdivia A. Kong M. Alvarez A. Cardenas A. Quest A. F. (2013). Thy-1-interacting molecules and cellular signaling in cis and trans. *Int. Rev. Cell Mol. Biol.* 305 163–216. 10.1016/B978-0-12-407695-2.00004-4 23890382

[B50] Hollrigel G. S. Morris R. J. Soltensz I. (1998). Enhanced inhibitory charge transfer during bursts of IPSCs in dentate granule cells in mice with regionally inhibited LTP. *Proc. R. Soc. Lond. B* 265 63–69. 10.1098/rspb.1998.0265 9470216 PMC1688757

[B51] Honerkamp-Smith A. R. Cicuta P. Collins M. D. Veatch S. L. den Nijs M. Schick M. (2008). Line tensions, correlation lengths, and critical exponents in lipid membranes near critical points. *Biophys. J.* 95 236–246. 10.1529/biophysj.107.128421 18424504 PMC2426649

[B52] Horejsi V. Zhang W. Schraven B. (2004). Transmembrane adaptor proteins: organizers of immunoreceptor signalling. *Nat. Rev. Immunol.* 4 603–616. 10.1038/nri1414 15286727

[B53] Hueber A. O. Bernard A. M. Battari C. L. Marguet D. Massol P. Foa C. (1997). Thymocytes in Thy-1-/- mice show augmented TCR signaling and impaired differentiation. *Curr. Biol.* 7 705–708. 10.1016/S0960-9822(06)00300-9 9285719

[B54] Hueber A. O. Pierres M. He H. T. (1992). Sulfated glycans directly interact with mouse Thy-1 and negatively regulate Thy-1-mediated adhesion of thymocytes to thymic epithelial cells. *J. Immunol.* 148 3692–3699. 1376338

[B55] Huttner W. B. Zimmerberg J. (2001). Implications of lipid microdomains for membrane curvature, budding and fission. *Curr. Opin. Cell Biol.* 13 478–484. 10.1016/S0955-0674(00)00239-8 11454455

[B56] Ipsen J. H. Karlstrom G. Mouritsen O. G. Wennerstrom H. Zuckermann M. J. (1987). Phase equilibria in the phosphatidylcholine-cholesterol system. *Biochim. Biophys. Acta* 905 162–172. 10.1016/0005-2736(87)90020-4 3676307

[B57] Kiessling V. Wan C. Tamm L. K. (2009). Domain coupling in asymmetric lipid bilayers. *Biochim. Biophys. Acta* 1788 64–71. 10.1016/j.bbamem.2008.09.003 18848518 PMC3099438

[B58] Kong M. Munoz N. Valdivia A. Alvarez A. Herrera-Molina R. (2013). Thy-1-mediated cell-cell contact induces astrocyte migration through the engagement of alphaVbeta3 integrin and syndecan-4. *Biochim. Biophys. Acta* 1833 1409–1420. 10.1016/j.bbamcr.2013.02.013 23481656 PMC3726046

[B59] Kumar A. Bhanja A. Bhattacharyya J. Jaganathan B. G. (2016). Multiple roles of CD90 in cancer. *Tumour. Biol.* 37 11611–11622. 10.1007/s13277-016-5112-0 27337957

[B60] Kuzmin P. I. Akimov S. A. Chizmadzhev Y. A. Zimmerberg J. Cohen F. S. (2005). Line tension and interaction energies of membrane rafts calculated from lipid splay and tilt. *Biophys. J.* 88 1120–1133. 10.1529/biophysj.104.048223 15542550 PMC1305117

[B61] Lagos-Cabre R. Alvarez A. Kong M. Burgos-Bravo F. Cardenas A. Rojas-Mancilla E. (2017). alphaVbeta3 Integrin regulates astrocyte reactivity. *J. Neuroinflamm.* 14:194. 10.1186/s12974-017-0968-5 28962574 PMC5622429

[B62] Lee I. H. Saha S. Polley A. Huang H. Mayor S. Rao M. (2015). Live cell plasma membranes do not exhibit a miscibility phase transition over a wide range of temperatures. *J. Phys. Chem. B* 119 4450–4459. 10.1021/jp512839q 25747462

[B63] Leyton L. Schneider P. Labra C. V. Ruegg C. Hetz C. A. Quest A. F. (2001). Thy-1 binds to integrin beta(3) on astrocytes and triggers formation of focal contact sites. *Curr. Biol.* 11 1028–1038. 10.1016/S0960-9822(01)00262-7 11470407

[B64] Lichtenberg D. Ahyayauch H. Goni F. M. (2013). The mechanism of detergent solubilization of lipid bilayers. *Biophys. J.* 105 289–299. 10.1016/j.bpj.2013.06.007 23870250 PMC3714928

[B65] Lichtenberg D. Goni F. M. Heerklotz H. (2005). Detergent-resistant membranes should not be identified with membrane rafts. *Trends Biochem. Sci.* 30 430–436. 10.1016/j.tibs.2005.06.004 15996869

[B66] Lin T. J. Lu K. W. Chen W. H. Cheng C. M. Lin Y. W. (2015). Roles of syndecan-4 and relative kinases in dorsal root ganglion neuron adhesion and mechanotransduction. *Neurosci. Lett.* 592 88–93. 10.1016/j.neulet.2015.02.058 25757361

[B67] Liu B. Chen W. Evavold B. D. Zhu C. (2014). Accumulation of dynamic catch bonds between TCR and agonist peptide-MHC triggers T cell signaling. *Cell* 157 357–368. 10.1016/j.cell.2014.02.053 24725404 PMC4123688

[B68] Liu X. Wong S. S. Taype C. A. Kim J. Shentu T. P. Espinoza C. R. (2017). Thy-1 interaction with Fas in lipid rafts regulates fibroblast apoptosis and lung injury resolution. *Lab. Invest.* 97 256–267. 10.1038/labinvest.2016.145 28165468 PMC5663248

[B69] Madore N. Smith K. L. Graham C. H. Jen A. Brady K. Hall S. (1999). Functionally different GPI proteins are organised in different domains on the neuronal surface. *EMBO J.* 18 6917–6926. 10.1093/emboj/18.24.6917 10601014 PMC1171755

[B70] Maldonado H. Calderon C. Burgos-Bravo F. Kobler O. Zuschratter W. Ramirez O. (2017). Astrocyte-to-neuron communication through integrin-engaged Thy-1/CBP/Csk/Src complex triggers neurite retraction via the RhoA/ROCK pathway. *Biochim. Biophys. Acta* 1864 243–254. 10.1016/j.bbamcr.2016.11.006 27842221

[B71] Mason D. W. Williams A. F. (1980). The kinetics and binding to membrane antigens in solution and at the cell surface. *Biochem. J.* 187 1–20. 10.1042/bj1870001 6967725 PMC1162489

[B72] Mayeux-Portas V. File S. E. Stewart C. L. Morris R. J. (2000). Mice lacking the cell adhesion molecule Thy-1 fail to use socially-transmitted cues to direct their choice of food. *Curr. Biol.* 10 68–75. 10.1016/S0960-9822(99)00278-X 10662668

[B73] Mayor S. Rothberg K. G. Maxfield F. R. (1994). Sequestration of GPI-anchored proteins in caveolae triggered by cross-linking. *Science* 264 1948–1951. 10.1126/science.7516582 7516582

[B74] Morris R. (1992). Thy-1, the enigmatic extrovert on the neuronal surface. *BioEssays* 14 715–722. 10.1002/bies.950141014 1285421

[B75] Morris R. Cox H. Mombelli E. Quinn P. J. (2004). Rafts, little caves and large potholes: how lipid structure interacts with membrane proteins to create functionally diverse membrane environments. *Subcell Biochem.* 37 35–118. 10.1007/978-1-4757-5806-1_2 15376618

[B76] Morris R. J. (1994). “Antigen-antibody interactions: how affinity and kinetics affect assay design and selection procedures,” in *Monoclonal Antibodies*, eds Ritter M. A. Ladyman H. (Cambridge: Cambridge University Press), 34–59.

[B77] Morris R. J. (2010). Ionic control of the metastable inner leaflet of the plasma membrane: fusions natural and artefactual. *FEBS Lett.* 584 1665–1669. 10.1016/j.febslet.2009.11.017 19913542

[B78] Morris R. J. Barber P. C. (1983). Fixation of Thy-1 in nervous tissue for immunohistochemistry: a quantitative assessment of the effect of different fixation conditions upon retention of antigenicity and the cross-linking of Thy-1. *J. Histochem. Cytochem.* 31 263–274. 10.1177/31.2.6131917 6131917

[B79] Morris R. J. Jen A. Warley A. (2011). Isolation of nano-meso scale detergent resistant membrane that has properties expected of lipid ‘rafts’. *J. Neurochem.* 116 671–677. 10.1111/j.1471-4159.2010.07076.x 21214574

[B80] Morris R. J. Nosten-Bertrand M. (1996). NOS and aggression. *Trends Neurosci.* 19 277–278. 10.1016/S0166-2236(96)20025-6 8799970

[B81] Munro S. (2003). Lipid rafts: elusive or illusive? *Cell* 115 377–388. 10.1016/S0092-8674(03)00882-1 14622593

[B82] Nemoto Y. L. Morris R. J. Hijikata H. Tsunoyama T. A. Shibata A. C. E. Kasai R. S. (2017). Dynamic meso-scale anchorage of GPI-anchored receptors in the plasma membrane: prion protein vs. Thy1. *Cell Biochem. Biophys.* 75 399–412. 10.1007/s12013-017-0808-3 28646414 PMC5691105

[B83] Nosten-Bertrand M. Errington M. L. Murphy K. P. S. J. Tokugawa Y. Barboni E. Kozlova E. (1996). Normal spatial learning despite regional inhibition of LTP in mice lacking Thy-1. *Nature* 379 826–829. 10.1038/379826a0 8587606

[B84] Parkyn C. J. Vermeulen E. G. Mootoosamy R. C. Sunyach C. Jacobsen C. Oxvig C. (2008). LRP1 controls biosynthetic and endocytic trafficking of neuronal prion protein. *J. Cell Sci.* 121 773–783. 10.1242/jcs.021816 18285446

[B85] Paulick M. G. Bertozzi C. R. (2008). The glycosylphosphatidylinositol anchor: a complex membrane-anchoring structure for proteins. *Biochemistry* 47 6991–7000. 10.1021/bi8006324 18557633 PMC2663890

[B86] Picke A. K. Campbell G. M. Bluher M. Krugel U. Schmidt F. N. Tsourdi E. (2018). Thy-1 (CD90) promotes bone formation and protects against obesity. *Sci. Transl. Med.* 10:eaao6806. 30089635 10.1126/scitranslmed.aao6806

[B87] Puig B. Altmeppen H. Glatzel M. (2014). The GPI-anchoring of PrP: implications in sorting and pathogenesis. *Prion* 8 11–18. 10.4161/pri.27892 24509692 PMC7030901

[B88] Raghupathy R. Anilkumar A. A. Polley A. Singh P. P. Yadav M. Johnson C. (2015). Transbilayer lipid interactions mediate nanoclustering of lipid-anchored proteins. *Cell* 161 581–594. 10.1016/j.cell.2015.03.048 25910209 PMC4651428

[B89] Rege T. A. Pallero M. A. Gomez C. Grenett H. E. Murphy-Ullrich J. E. Hagood J. S. (2006). Thy-1, via its GPI anchor, modulates Src family kinase and focal adhesion kinase phosphorylation and subcellular localization, and fibroblast migration, in response to thrombospondin-1/hep I. *Exp. Cell Res.* 312 3752–3767. 10.1016/j.yexcr.2006.07.029 17027000

[B90] Shentu T. P. Huang T. S. Cernelc-Kohan M. Chan J. Wong S. S. Espinoza C. R. (2017). Thy-1 dependent uptake of mesenchymal stem cell-derived extracellular vesicles blocks myofibroblastic differentiation. *Sci. Rep.* 7:18052. 10.1038/s41598-017-18288-9 29273797 PMC5741716

[B91] Shibata A. C. Fujiwara T. K. Chen L. Suzuki K. G. Ishikawa Y. Nemoto Y. L. (2012). Archipelago architecture of the focal adhesion: membrane molecules freely enter and exit from the focal adhesion zone. *Cytoskeleton (Hoboken)* 69 380–392. 10.1002/cm.21032 22488960

[B92] Sibener L. V. Fernandes R. A. Kolawole E. M. Carbone C. B. Liu F. McAffee D. (2018). Isolation of a structural mechanism for uncoupling T cell receptor signaling from peptide-MHC binding. *Cell* 174 672.e27–687.e27. 10.1016/j.cell.2018.06.017 30053426 PMC6140336

[B93] Storck E. M. Ozbalci C. Eggert U. S. (2018). Lipid cell biology: a focus on lipids in cell division. *Annu. Rev. Biochem.* 87 839–869. 10.1146/annurev-biochem-062917-012448 29494237

[B94] Sudhof T. C. (2013). Neurotransmitter release: the last millisecond in the life of a synaptic vesicle. *Neuron* 80 675–690. 10.1016/j.neuron.2013.10.022 24183019 PMC3866025

[B95] Sunyach C. Jen A. Deng J. Fitzgerald K. Frobert Y. McCaffrey M. W. (2003). The mechanism of internalisation of GPI anchored prion protein. *EMBO J.* 22 3591–3601. 10.1093/emboj/cdg344 12853474 PMC165614

[B96] Surviladze Z. Harrison K. A. Murphy R. C. Wilson B. S. (2007). FcepsilonRI and Thy-1 domains have unique protein and lipid compositions. *J. Lipid Res.* 48 1325–1335. 10.1194/jlr.M600485-JLR200 17387221

[B97] Suzuki K. G. Fujiwara T. K. Edidin M. Kusumi A. (2007a). Dynamic recruitment of phospholipase C gamma at transiently immobilized GPI-anchored receptor clusters induces IP3-Ca2+ signaling: single-molecule tracking study 2. *J. Cell Biol.* 177 731–742. 10.1083/jcb.200609175 17517965 PMC2064217

[B98] Suzuki K. G. Fujiwara T. K. Sanematsu F. Iino R. Edidin M. Kusumi A. (2007b). GPI-anchored receptor clusters transiently recruit Lyn and G alpha for temporary cluster immobilization and Lyn activation: single-molecule tracking study 1. *J. Cell Biol.* 177 717–730. 10.1083/jcb.200609174 17517964 PMC2064216

[B99] Suzuki K. G. Kasai R. S. Hirosawa K. M. Nemoto Y. L. Ishibashi M. Miwa Y. (2012). Transient GPI-anchored protein homodimers are units for raft organization and function. *Nat. Chem. Biol.* 8 774–783. 10.1038/nchembio.1028 22820419

[B100] Suzuki K. G. N. Ando H. Komura N. Konishi M. Imamura A. Ishida H. (2018). Revealing the raft domain organization in the plasma membrane by single-molecule imaging of fluorescent ganglioside analogs. *Methods Enzymol.* 598 267–282. 10.1016/bs.mie.2017.06.038 29306438

[B101] Tanimura N. Saitoh S. Kawano S. Kosugi A. Miyake K. (2006). Palmitoylation of LAT contributes to its subcellular localization and stability. *Biochem. Biophys. Res. Commun.* 341 1177–1183. 10.1016/j.bbrc.2006.01.076 16460687

[B102] Tian T. Harding A. Inder K. Plowman S. Parton R. G. Hancock J. F. (2007). Plasma membrane nanoswitches generate high-fidelity Ras signal transduction. *Nat. Cell Biol.* 9 905–914. 10.1038/ncb1615 17618274

[B103] Tilcock C. P. Cullis P. R. (1981). The polymorphic phase behaviour of mixed phosphatidylserine-phosphatidylethanolamine model systems as detected by 31P-NMR. *Biochim. Biophys. Acta* 641 189–201. 10.1016/0005-2736(81)90583-6 7194114

[B104] Tiveron M. C. Barboni E. Pliego Rivero F. B. Gormley A. M. Seeley P. J. Grosveld F. (1992). Selective inhibition of neurite outgrowth on mature astrocytes by Thy-1 glycoprotein. *Nature* 355 745–748. 10.1038/355745a0 1346926

[B105] Tokutomi S. Lew R. Ohnishi S. (1981). Ca2+-induced phase separation in phosphatidylserine, phosphatidylethanolamine and phosphatidylcholine mixed membranes. *Biochim. Biophys. Acta* 643 276–282. 10.1016/0005-2736(81)90073-0 6261813

[B106] Tomaselli K. J. Doherty P. Emmett C. J. Damsky C. H. Walsh F. S. Reichardt L. F. (1993). Expression of beta 1 integrins in sensory neurons of the dorsal root ganglion and their functions in neurite outgrowth on two laminin isoforms. *J. Neurosci.* 13 4880–4888. 10.1523/JNEUROSCI.13-11-04880.1993 7693896 PMC2710121

[B107] Tsunoyama T. A. Watanabe Y. Goto J. Naito K. Kasai R. S. Suzuki K. G. N. (2018). Super-long single-molecule tracking reveals dynamic-anchorage-induced integrin function. *Nat. Chem. Biol.* 14 497–506. 10.1038/s41589-018-0032-5 29610485

[B108] Uversky V. N. (2017). Intrinsically disordered proteins in overcrowded milieu: membrane-less organelles, phase separation, and intrinsic disorder. *Curr. Opin. Struct. Biol.* 44 18–30. 10.1016/j.sbi.2016.10.015 27838525

[B109] Valitutti S. Muller S. Cella M. Padovan E. Tiveron M. C. (1995). Serial triggering of many T-cell receptors by a few peptide-MHC complexes. *Nature* 375 148–151. 10.1038/375148a0 7753171

[B110] van’t Hof W. Resh M. D. (2000). Targeting proteins to plasma membrane and membrane microdomains by N-terminal myristoylation and palmitoylation. *Methods Enzymol.* 327 317–330. 10.1016/S0076-6879(00)27287-X 11044994

[B111] Varisco B. M. Ambalavanan N. Whitsett J. A. Hagood J. S. (2012). Thy-1 signals through PPARgamma to promote lipofibroblast differentiation in the developing lung. *Am. J. Respir. Cell Mol. Biol.* 46 765–772. 10.1165/rcmb.2011-0316OC 22268140 PMC3380285

[B112] Veatch S. L. Chiang E. N. Sengupta P. Holowka D. A. Baird B. A. (2012). Quantitative nanoscale analysis of IgE-FcepsilonRI clustering and coupling to early signaling proteins. *J. Phys. Chem. B* 116 6923–6935. 10.1021/jp300197p 22397623 PMC3376227

[B113] Wandel E. Saalbach A. Sittig D. Gebhardt C. Aust G. (2012). Thy-1 (CD90) is an interacting partner for CD97 on activated endothelial cells. *J. Immunol.* 188 1442–1450. 10.4049/jimmunol.1003944 22210915

[B114] Wetzel A. Chavakis T. Preissner K. T. Sticherling M. Haustein U. F. Anderegg U. (2004). Human Thy-1 (CD90) on activated endothelial cells is a counterreceptor for the leukocyte integrin Mac-1 (CD11b/CD18). *J. Immunol.* 172 3850–3859. 10.4049/jimmunol.172.6.3850 15004192

[B115] Williams A. F. Barclay A. N. Letarte-Muirhead M. Morris R. J. (1977). The tissue distribution, purification and chemical composition of the rat Thy-1 antigen. *Cold Spring Harbor. Symp. Quant. Biol.* 49 51–61. 10.1101/SQB.1977.041.01.009 70317

[B116] Zhou Y. Hagood J. S. Lu B. Merryman W. D. Murphy-Ullrich J. E. (2010). Thy-1-integrin alphav beta5 interactions inhibit lung fibroblast contraction-induced latent transforming growth factor-beta1 activation and myofibroblast differentiation. *J. Biol. Chem.* 285 22382–22393. 10.1074/jbc.M110.126227 20463011 PMC2903374

[B117] Zhou Y. Hagood J. S. Murphy-Ullrich J. E. (2004). Thy-1 expression regulates the ability of rat lung fibroblasts to activate transforming growth factor-beta in response to fibrogenic stimuli. *Am. J. Pathol.* 165 659–669. 10.1016/S0002-9440(10)63330-5 15277239 PMC1618578

